# Psychometric properties of a Creole version of Medical Outcome Study – Short Form 36 among type II diabetes patients on Reunion Island

**DOI:** 10.3389/fpubh.2023.1186153

**Published:** 2023-08-21

**Authors:** Ibtissame Soulaimana, Léa Bruneau, Antoine Tisseaux, Maissa Safieddine, Xavier Debussche, Sophie Lafarge, Bruno Falissard, Cyril Ferdynus, Laetitia Huiart

**Affiliations:** ^1^Department of Public Health and Research Support, Methodological Support and Biostatistics, Centre Hospitalier de la Réunion, Saint Denis, France; ^2^Santé Publique France, Mamoudzou, Mayotte, France; ^3^Inserm CIC 1410, Centre Hospitalier Universitaire de La Réunion, Saint Pierre, France; ^4^Department of Endocrinology, Centre Hospitalier Universitaire de la Réunion, Saint Denis, France; ^5^INSERM U1153 Centre de Recherche Épidémiologie et Statistique, Paris, France; ^6^Santé Publique France, Saint Maurice, France

**Keywords:** quality of life, SF-36, Creole-translation, psychometric properties, type II diabetes, Reunion Island

## Abstract

**Introduction:**

Health quality of life assessment is particularly important to measure the impact of chronic diseases. The aims of this study were to provide a cross-culturally adapted Creole-translation of the Medical Outcome Study Short-Form 36 (SF-36) and to assess psychometric performance of the Creole and French versions of the SF-36 among patients with type II diabetes in Reunion Island.

**Materials and methods:**

The Creole translation and cross-cultural adaptation processes were based on the International Quality Of Life Assessment (IQOLA) methods. Internal consistency, test–retest reliability, convergent and discriminant validity using Multi-Trait-Multi-Method analysis and structural validity using exploratory factor analysis of the SF-36 for both versions were performed.

**Results:**

In the Creole version of the SF-36, Cronbach’s alpha exceeded 0.70 for all subscales except general health. In the French SF-36, Cronbach’s alpha exceeded 0.70 on all subscales except general health and bodily pain. In the Creole SF-36, intraclass correlation coefficient (ICC) for reproducibility was suboptimal. Multi-trait multi-method analysis showed that item-scale correlation exceeded 0.4 for all items except two general health items of the Creole SF-36 and one of the French SF-36. Factor analysis of 2 versions showed that the physical functioning, vitality, and mental health were each divided into two subscales.

**Discussion:**

Overall, our findings provided evidence that the SF-36 is adapted to Reunion Island in both Creole and French versions. However, further research could be conducted to investigate French–Creole differences in perceived health status and a cultural adaptation of the French version will be considered.

## Introduction

Reunion Island is a French oversea territory located in the Indian Ocean, where ethnic diversity is important. Its population is mostly characterized by a cultural and religious heterogenicity and both Creole and French languages are spoken. Creole is mainly derived from French but includes many terms from other languages (Malagasy, Hindi, Portuguese, Gujarati and Tamil) (1). In Reunion Island, Creole is a language mostly spoken and rarely written and there is no single graphic system recognized for its writing.

Quality of life is increasingly used as an outcome for the assessment of health care interventions (2). As a consequence, the measurement of health status has become a crucial issue (3). Even though Reunion Island is a French territory, the local specificities may have an impact on the measurement properties of generic psychometric tools, like the Medical Outcomes Study (MOS) 36-Item Short-Form Health Survey (SF-36). SF-36 is one of the generic tools measuring health-related quality of life (HRQoL). This is a generic scale suitable for any patient regardless of health status (4, 5). It is a reference instrument designed for use in clinical practice and research, health policy evaluations, and general population surveys (6). International interest regarding this questionnaire has led to its translation in more than 60 countries (7, 8), including France (9). However, it is questionable whether the French version of the SF-36 (9) is adapted to the specific population of Reunion Island.

In Reunion Island, type II diabetes is a major public health problem, with a prevalence of 10%. Standardized incidence rates are higher in Reunion Island than in mainland France for certain types of diabetic complications, such as dialysis or kidney transplants (218/100000 diabetics treated) or strokes (675/100000) (10). As this chronic disease has an impact on quality of life (11), its accurate evaluation is necessary in this population. The lack of validated psychometric measurement tools in Reunion Island’s population, has been one of the main difficulties in conducting clinical and epidemiological research to measure quality of life.

Thus, the aim of this study was to translate and adapt a Creole version of SF-36 and to evaluate its psychometric properties on patients with type II diabetes in Reunion Island. We also aimed to evaluate the psychometric properties of the French version of SF-36 in this population.

## Materials and methods

### Design and sampling

The first step was to translate and culturally adapt the French SF-36 (Version 1) into Creole (step not involving the human person). The second step was a cross-sectional study to validate the Creole version among 148 diabetic patients. We also took the opportunity to validate the French SF-36 version in another independent sampling of 152 Reunionese diabetic patients, with a cross-sectional study.

Participants were a convenience sample of diabetic patients who consulted for follow-up of their diabetes in two diabetology departments of the University Hospital of Reunion Island (Saint-Denis and Saint-Pierre). All included patients were over 18 years of age, diagnosed with type II diabetes for at least 1 year, proficient in speaking Creole and living in Reunion Island for at least 5 years. Exclusion criteria were patients unable to understand and comply with the study procedures, with cognitive impairment, a history of stroke with neurological or motor disability, or severe acute complications of diabetes.

The sample size was calculated according to the criteria required for the Factorial Analysis technique (i.e., between four and five subjects per item), considering the number of items in the scale (12). Thus, a sample of at least 148 participants was considered adequate, giving us a statistical power of 87% to show a difference between a Cronbach’s alpha equal to 0.70 under the null hypothesis, and equal to 0.80 under the alternative hypothesis, for the SF-36 scale.

### Ethical considerations

Before inclusion, the aim of the study was explained to participants, who then filled an informed consent form.36-item short form.

### Instrument

The SF-36 Health Survey is a generic health status measurement instrument which contains 36 questions. It consists of 8 subscales assessing several physical and mental dimensions: Physical Functioning (PF), Role limitations due to Physical health problems (RP), Bodily Pain (BP), General Health perceptions (GH), Vitality (VT), Social Functioning (SF), Role limitations due to Emotional problems (RE) and Mental Health (MH). An additional item is assessing perceived change over the past 12 months (out of the eight subscales). The responses are used to calculate a score ranging from 0 to 100 with higher score representing better health status (13).

### Translation and cultural adaptation methods

**Figure 1:**
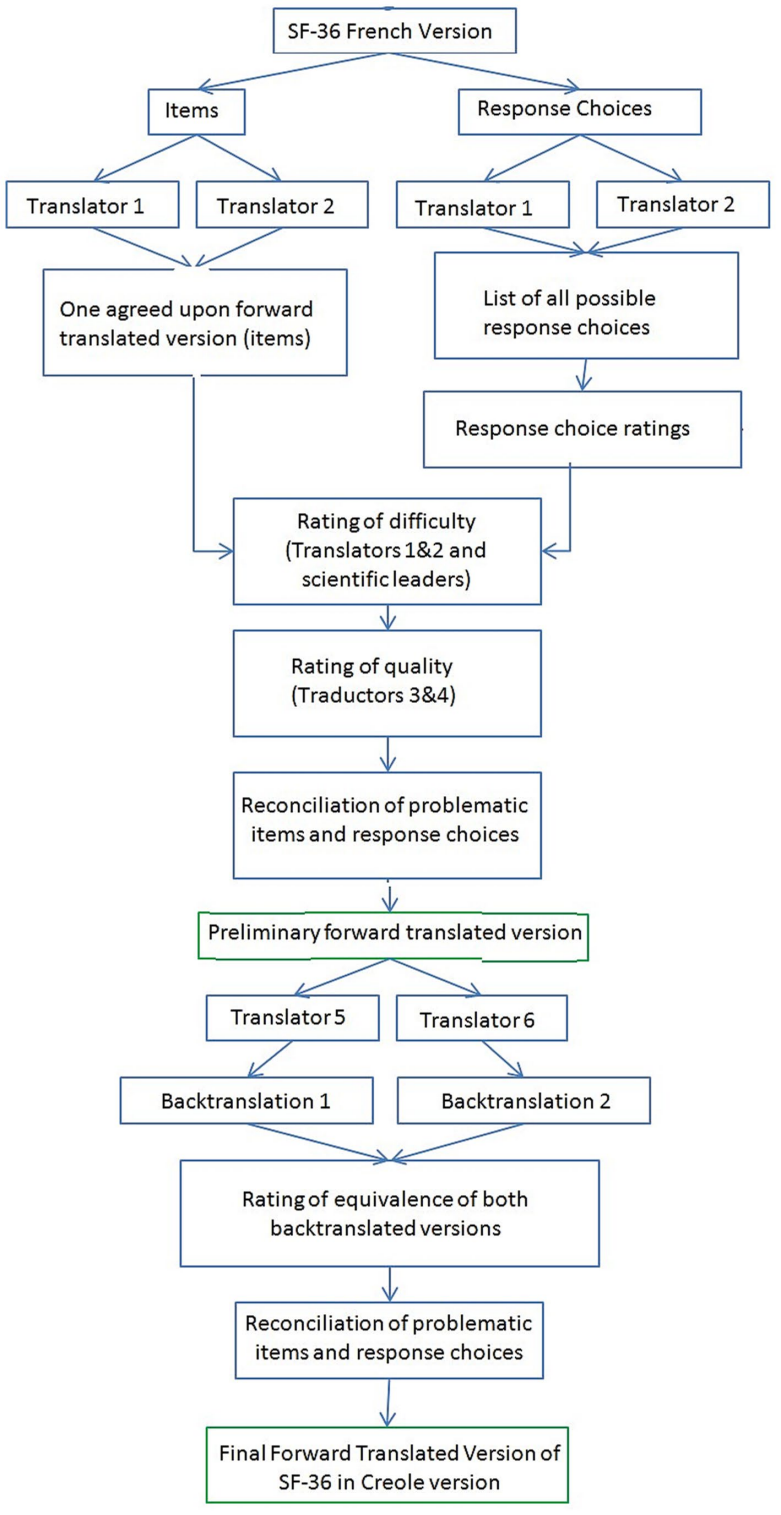
SF-36 translation procedure.

Translation and cross-cultural adaptation processes were in accordance with The International Quality of Life Assessment (IQOLA) methods (14) (Figure 1). Because Creole is mainly derived from French, we chose the French SF-36 questionnaire as the original version for adaptation and translation to Creole.

#### Step 1

After authorization from IQOLA, the French SF-36 was adapted and translated into Creole two independent translators who were native speakers of the target language (Creole language) and fluent in French. This translation was combined with a cultural adaptation of items and response choices. Both were advised that the translation should be semantic rather than literal to attain conceptual and linguistic equivalences. The translators were also asked to give different translations for each response choice where possible.

Translators rated the difficulty of translating each item and response choices using a rating scale ranging from 0 (not at all difficult) to 100 (extremely difficult) and provided comments where appropriate (14). The extent of agreement between raters was evaluated by calculating the percentage of ratings for which raters agreed within 0 to 15 points, on a 100-point scale (14).

After review by an expert committee, composed of two linguistic experts, two translators and on expert researcher in psychometric scales, the preliminary version was edited and sent to IQOLA group to obtain their agreement to proceed to the next step.

#### Step 2

This first version was then translated back into French by two independent bilingual translators, both French native speakers and fluent in Creole. These translators also rated the quality of the original translation (clarity) on a scale from 0 (not at all perfect) to 100 (perfect). Backtranslations of SF-36 Creole were reviewed by the expert committee.

#### Step 3

The pre-final version was edited and tested on a population of 10 diabetics patients. Each subject had to answer the questionnaire and was asked about difficulties in understanding the items, in order to identify inconsistencies that were not raised by the experts.

The final version of the SF-36 in Creole was then created thanks to the information obtained in the previous steps and approved by the expert committee.

### Setting and data collection

Patients self-completed the questionnaire, or were helped by a Creole-speaker technician, to not exclude illiterate populations. There is a high proportion of illiteracy in Reunion (23%).

Additional data were collected in a socio-demographic and clinical questionnaire.

### Statistical analysis

Validity assessment of the SF-36 in Creole and French followed the IQOLA Project research protocol. According to the IQOLA project, the validity of health questionnaires has most often been evaluated using content, construct, and criterion validity (15). Analyses of psychometric properties were performed independently for Creole and French version.

Floor and ceiling effects for all items were assessed using the number and percentage of total patients with the lowest and highest possible scores, which should be less than 15% (9). After calculating the score for each dimension, according to the IQOLA methodology, distribution of the 8 subscales was assessed. Mean, standard deviation, median, first and third quartiles, lowest and highest scores were presented.

#### Reliability

Internal consistency was measured for the overall questionnaire and individually for each of the 8 subscales using Cronbach’s alpha coefficient. A Cronbach’s alpha of 0.70 or higher was considered satisfactory (16).

Reliability was also assessed using intra-class correlation coefficients (ICC) and their 95% confidence intervals (CI) between SF-36 scores after a test–retest procedure. The retest procedure was conducted on a random sample of 27 patients, 1 month after inclusion, for the Creole version only. An ICC >0.5 was considered acceptable and an ICC > 0.7 was considered as good (17).

#### Validity

Convergent and discriminant validity were assessed using a multi-trait-multi-method analysis (MTMM) (18). This technique based on an examination of item-scale correlations, was employed to confirm the hypothesized scale structure of the SF-36. Two criteria were used: item convergent validity which was sustained when item-scale correlations were 0.40 or above; and item discriminant validity which was sustained when correlation between a specific item was significantly higher with its own scale than with the other scales.

Exploratory factor analysis (EFA) with varimax rotation was carried out to examine structural validity (19). EFA was used because there was no prior knowledge or theory regarding the underlying factor structure of the SF-36 on diabetics creolophone population. Given the particular cross-cultural context of the study, we wanted to know the extent to which the structure deviated or not from the original structure. The Kaiser–Meyer–Olkin (KMO) test was calculated to determine the validity of this factor analysis. The appropriate number of factors was determined based on the screen plot, as originally proposed by Cattell (12).

Statistical analyses were performed using R version 3.5.3 with packages psy (v1.1), psych (v1.8.12), ltm (v1.1–1), boot (v1.3–22).

## Results

### Translation and cultural adaptation of the SF-36 into Creole

By Strictly following the procedure detailed above, the SF-36 was translated into Creole language without major difficulty. The average difficulty of the 36 items was 16.1 (SD = 18.9). Nine items (25.0%) and 8 responses (40.0%) received an average difficulty ratings greater than 25 (14). The percentage of inter-rater agreement (between two raters) was 70.0% for the difficulty of the items and 64.3% for the difficulty of the responses. All items and responses received clarity ratings below 80 (14). Median item clarity was 40.0 (IQR: 37.5–45.0) and median response clarity was 70.0 (IQR: 57.5–70.0). The inter-rater agreement was 58.0% for item clarity and 60.0% for response clarity.

### Patient characteristics and score distribution

Validation of SF-36 in Creole and French languages was conducted on two independent samples including 148 and 152 subjects, respectively, in each group. All questionnaires in the Creole version were hetero-administrated. For the French version, 146 (96.1%) were hetero-administrated and 6 (3.9%) were self-administrated. Socio-demographic and clinical data are reported in Table 1. The mean age was 57.0 (SD = 10.5) years for the Creole version and 56.0 (SD = 11.9) years for the French version. The mean time since Type 2 diabetes diagnosis was 16.0 (SD = 10.6) years for the Creole version sample and 17.0 (SD = 13.9) years for the French version sample.

**Table 1:** Socio-demographic and clinical characteristics of patients.

Socio-demographic variables	SF-36 Creole version (*N* = 148)	SF-36 French version (*N* = 152)
Age (years) [*n* (%)]		
21–49	30 (20.3%)	34 (22.4%)
50–64	87 (58. 8%)	77 (50.7%)
65 +	31 (20.9%)	40 (26.3%)
Missing	0	1 (0.7%)
Gender [*n* (%)]		
Female	77 (52.1%)	88 (57.9%)
Male	71 (47.9%)	64 (42.1%)
Professional status [*n* (%)]		
Employed	86 (58.1%)	85 (55.9%)
Unemployed	62 (41.9%)	66 (43.4%)
Missing	0	1 (0.7%)
Education level [*n* (%)]		
Out-of-school	5 (3.4%)	8 (5.3%)
Elementary school	52 (35.1%)	43 (28.3%)
Middle school	42 (28.4%)	42 (27.6%)
High school	31 (20.9%)	29 (19.1%)
College and above	16 (10.8%)	24 (15.8%)
Other	2 (1.4%)	5 (3.3%)
Missing	0	1 (0.7%)
Duration of diabetes		
Between 1 and 2 years	9 (6.1%)	4 (2.6%)
Between 2 and 5 years	11 (7.4%)	11 (7.2%)
5 years and above	109 (73.7%)	87 (57.2%)
Missing	19 (12.8%)	50 (32.9%)
BMI (kg/m^2^)		
Normal	18 (12.2%)	20 (13.2%)
Overweight	42 (28.4%)	38 (25.0%)
Obese I	37 (25.0%)	36 (23.7%)
Obese II	16 (10.8%)	19 (12.5%)
Obese III	10 (6.8%)	13 (8.6%)
Missing	25 (16.9%)	26 (17.1%)
Diabetes complications		
Severe foot injury	10 (9.7%)	8 (6.7%)
Retinopathy	17 (16.5%)	18 (15.1%)
Comorbities		
Acute coronary accident	8 (7.8%)	25 (21.1%)
Ischemic Stroke	5 (4.8%)	13 (10.9%)
Angor	3 (2.9%)	6 (5.0%)
Coronary disease	10 (9.7%)	11 (9.2%)
Heart failure	1 (1.0%)	6 (5.0%)
High blood pressure	80 (77.7%)	101 (84.9%)
Neuropathy of lower limb	24 (23.3%)	35 (29.4%)
Arteriopathy	9 (8.7%)	11 (9.2%)
Macro-proteinuria	15 (14.6%)	24 (20.2%)

No floor or ceiling effects were observed in each item or each domain for either version. Table 2 shows the description of the 8 subscales of the SF-36 for both versions. For the Creole version, the mean score ranged from 55.5 (SD = 20.0) for the general health subscale to 69.3 (SD = 26.0) for physical functioning subscale. For the French version, the mean score ranged from 50.2 (SD = 19.2) for the vitality subscale to 68.5 (SD = 27.3) for physical functioning subscale.

**Table 2:** Distribution of scores and floor and ceiling effect of the SF-36 eight subscales.

Subscale	PF	RP	BP	GH	VT	SF	RE	MH	HT
Number of items	10	4	2	5	4	2	5	3	1
Creole version (*N* = 148)									
Mean score	69.3	64.2	64.1	55.5	56.3	75.0	68.4	61.0	56.6
Standard deviation	26.0	35.0	30.4	20.0	21.9	25.7	32.6	22.3	23.0
Median	75.0	75.0	68.9	50.0	55.0	75.0	75.0	60.0	50.0
First quartile	53.8	35.9	45.6	40.0	42.5	62.5	50.0	46.0	50.0
Third quartile	90.0	100.0	93.3	70.0	72.5	100.0	100.0	78.0	75.0
Percentage at ceiling	0.1	0.3	0.2	0.0	0.0	0.4	0.4	0.1	0.1
Percentage at floor	0.0	0.1	0.0	0.0	0.0	0.0	0.1	0.0	0.0
French version (*N* = 152)									
Mean score	68.5	65.5	61.0	53.4	50.2	73.6	65.2	58.8	58.9
Standard deviation	27.3	28.9	26.1	21.0	19.2	26.3	29.9	21.3	26.4
Median	75.0	68.8	57.8	55.0	50.0	75.0	66.7	58.0	50.0
First quartile	50.0	50.0	45.6	40.0	39.3	59.3	50.0	44.0	50.0
Third quartile	90.0	93.5	82.2	70.0	60.0	100.0	100.0	72.5	75.0
Percentage at ceiling	0.1	0.2	0.1	0.0	0.0	0.3	0.3	0.0	0.1
Percentage at floor	0.0	0.0	0.0	0.0	0.0	0.0	0.0	0.0	0.1

PF, physical function; RP, role-physical; BP, bodily pain; GH, general health; VT, vitality; SF, social functioning; RE, role-emotional; MH, mental health; HT, health transition.

### Evaluation of the psychometric properties of the SF-36 in Creole and French version

#### Internal consistency and inter-scale correlation.

Overall Cronbach’s alpha coefficient was 0.93 for both versions. All 8 subscales showed good internal consistency in this population, with Cronbach’s alpha for each dimension ranging from 0.63 (General health) to 0.95 (Role-physical) for Creole version and 0.61 (Bodily pain) to 0.91 (Physical functioning) for the French version (Table 3).

**Table 3:** Internal consistency and test–retest reliability.

	Cronbach’s coefficient		Intra-class correlation coefficient (95% CI)
Subscale	SF-36 Creole version (*N* = 148)	SF-36 French version (*N* = 152)	SF-36 Creole version (*N* = 27)
Physical function (PF)	0.90	0.91	0.79 (0.64–0.94)
Role physical (RP)	0.95	0.89	0.59 (0.21–0. 82)
Bodily pain (BP)	0.81	0.61	0.78 (0.57–0.91)
General health (GH)	0.63	0.68	0.61 (0.19–0.82)
Vitality (VT)	0.71	0.72	0.51 (0.08–0.79)
Social functioning (SF)	0.75	0.73	0.21 (0.00–0.61)
Mental health (MH)	0.74	0.79	0.57 (0.06–0.84)
Role emotional (RE)	0.94	0.90	0.46 (0.06–0.77)

Inter-scale correlations for Creole version ranged from 0.21 (between mental health and physical functioning) to 0.50 (between mental health and vitality; Table 4). Highest correlations were observed between mental health, vitality, social functioning, and role limitations due to emotional component. For the French version, inter-scale correlations ranged from 0.22 (between general health and physical functioning) to 0.49 (between role physical and role emotional).

**Table 4:** Correlation among the eight subscales of the SF-36.

		Subscales correlations						
Subsacle	Inter item correlation (range)^a^	PF	RP	BP	GH	VT	SF	MH
SF-36 Creole version								
Physical function (PF)	0.28–0.90							
Role physical (RP)	0.82–0.85	0.38						
Bodily pain (BP)	0.69	0.28	0.38					
General health (GH)	0.06–0.55	0.23	0.30	0.29				
Vitality (VT)	0.29–0.49	0.30	0.43	0.38	0.41			
Social functioning (SF)	0.60	0.22	0.34	0.24	0.27	0.33		
Mental health (MH)	0.17–0.54	0.21	0.34	0.29	0.38	0.50	0.48	
Role emotional (RE)	0.82–0.85	0.34	0.61	0.27	0.32	0.41	0.43	0.43
SF-36 French version								
Physical function (PF)	0.28–0.81							
Role physical (RP)	0.56–0.73	0.42						
Bodily pain (BP)	0.44	0.25	0.32					
General health (GH)	0.16–0.43	0.22	0.25	0.27				
Vitality (VT)	0.19–0.49	0.36	0.35	0.26	0.33			
Social functioning (SF)	0.58	0.24	0.27	0.26	0.27	0.32		
Mental health (MH)	0.28–0.62	0.31	0.26	0.22	0.31	0.45	0.45	
Role emotional (RE)	0.70–0.79	0.45	0.49	0.26	0.30	0.42	0.43	0.46

a Range of correlations (Pearson’s) between items on same subscale.

#### Test–retest reliability

A retest was carried out on 27 subjects included 1 month after the filling of the first questionnaire. Table 3 shows intra-class correlation coefficients of each of the 8 subscales. All Intra-class correlation coefficients were moderate to good (ranged from 0.51 to 0.79) except for RE (0.46, 95% CI 0.06–0.77) and SF (0.21, 95% CI 0.00–0.61).

#### Convergent and discriminant validity

In the Creole version, MTMM showed that all item-scale correlations exceeded 0.40, indicating good convergent validity, except for two items of ‘general health’ (GH) subscale: GH2 and GH3 who correlated at 0.27 and 0.35, respectively, with their own scale. In the French version, all item-scale correlations exceeded 0.40 except for one item (GH3) which had a correlation of 0.31 with its own scale (Table 5).

**Table 5:** Item convergent and discriminant validity (multi-trait multi-method).

	SF-36 Creole version (*N* = 148)		SF-36 French version (*N* = 152)	
Subscale	Correlations of Item with own scale (range)^a^	Correlations of Item with other scale (range)^b^	Correlations of Item with own scale (range)^a^	Correlations of Item with other scale (range)^b^
Physical function (PF)	0.52–0.73	0.05–0.42	0.47–0.77	0.13–0.57
Role physical (RP)	0.78–0.88	0.33–0.72	0.73–0.77	0.27–0.58
General health (GH)	0.27–0.49	0.08–0.47	0.30–0.56	0.08–0.41
Vitality (VT)	0.46–0.53	0.20–0.58	0.42–0.58	0.15–0.59
Social functioning (SF)	0.60–0.60	0.20–0.57	0.58–0.58	0.24–0.56
Role emotional (RE)	0.82–0.96	0.33–0.72	0.79–0.67	0.19–0.56
Mental health (MH)	0.42–0.62	0.09–0.59	0.44–0.68	0.50–0.56

a Range of correlations (Pearson’s) between items and hypothesized scale.

b Range of correlations (Pearson’s) between items and other scales.

Moreover, MTMM analysis showed that all items in the Creole and French versions had good discriminant validity. Scores for each item were generally significantly more closely correlated with their own scale than with the other scales.

However, for Creole version, GH1 («In general, would you say your health is:»), MH3 («Have you felt calm and peaceful?»), and MH5 («Have you been a happy person?») were more correlated with the ‘vitality’ (VT) subscale. VT2 («Did you have a lot of energy?») was more correlated with ‘mental health’ (MH) and GH subscales than with its own scale. For French version VT3 («Did you feel worn out?») and VT4 («Did you feel tired?») were more correlated with MH subscale and MH4 («Have you felt downhearted and blue?») was more correlated with VT subscale. All correlations are presented in Table 5.

#### Structural validity

**Figure 2:**
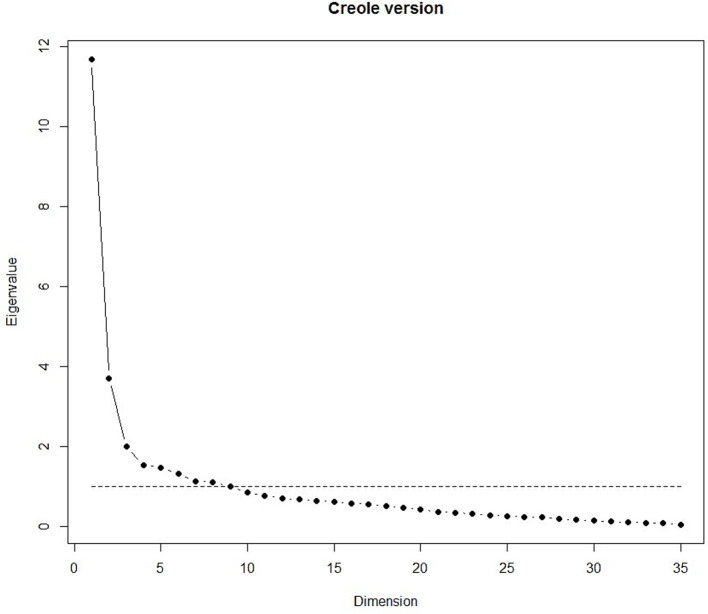
Scree plot of the Creole version.

**Figure 3:**
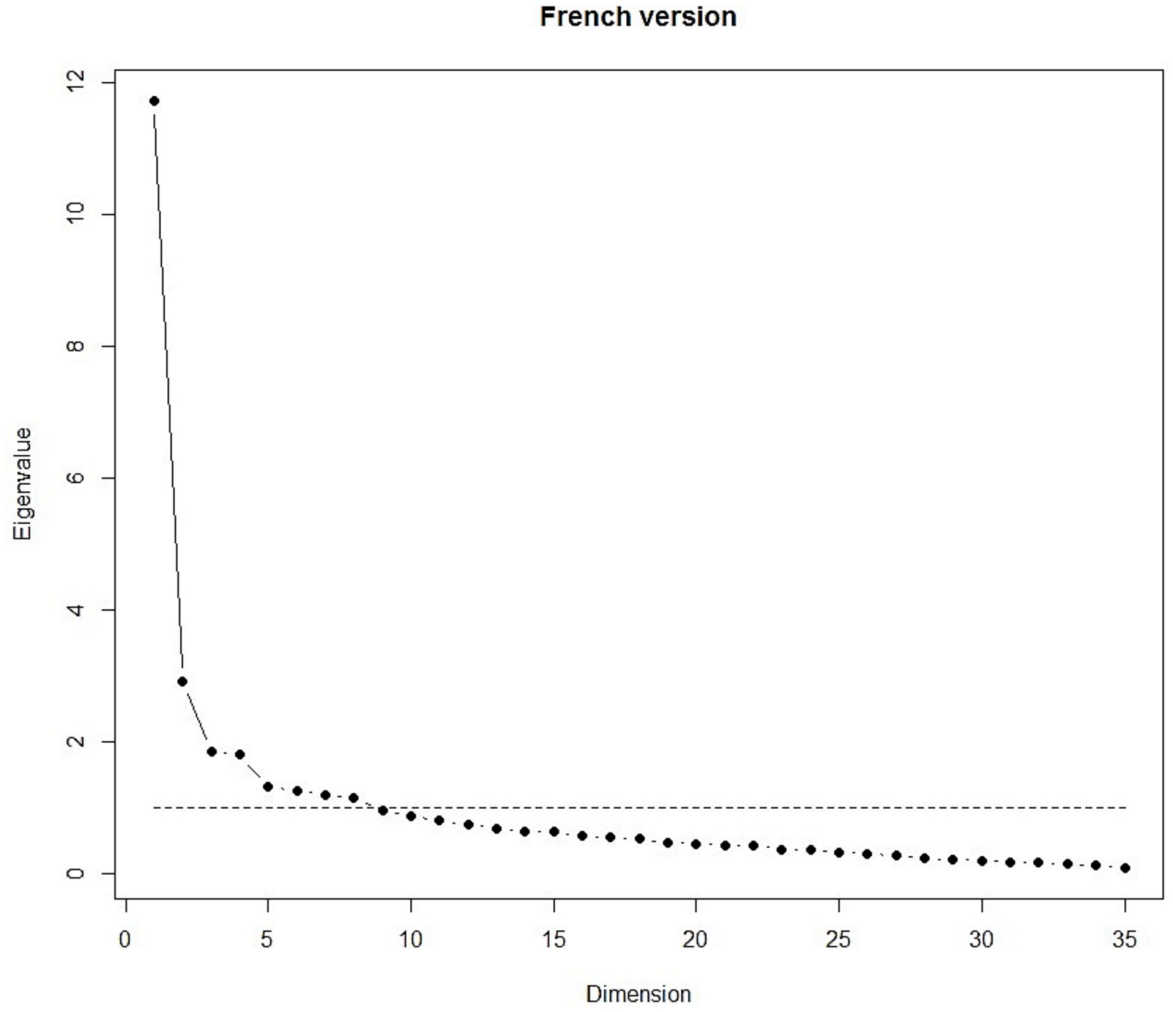
Scree plot of the French version.

Both models were suitable for factorial validity, with a Kaiser–Meyer–Olkin (KMO) value of 0.88. The scree plot, presented in Figures 2, 3, showed 8 eigenvalues greater than 1 for both versions.

Table 6 presents results of EFA with varimax rotation for the eight-factor model of the Creole version which accounted for 60.0% of the common variance. All items of the ‘Role Physical’ (RP), ‘Role Emotional’ (RE) and ‘Bodily Pain’ (BP) subscales each constituted a single factor (resp. 2, 6 and 7) showing good structural homogeneity. The ‘Physical Function’ subscale was divided into 2 factors (resp. 1 and 4). ‘Social Functioning’ (SF) and all but 3 items (resp. VT1, VT2, MH5) of ‘Mental Health’ (MH) and ‘Vitality’ (VT) subscales were constituted a unique factor, the factor 3. These 3 specific items were regrouped into factor 5. The ‘General Health’ (GH) subscale was heterogeneous and divided into 4 factors. GH1 had the highest loading on Factor 5 with ‘vitality’ (VT), GH2 on Factor 3 with and ‘Mental Health’ (MH), GH4 on Factor 2 with RP. GH3 and GH5 were grouped in the last factor, factor 8.

**Table 6:** Factor loadings from the exploratory factor analysis on the SF-36 Creole version.

	Factor1	Factor2	Factor3	Factor4	Factor5	Factor6	Factor7	Factor8
PF1	0.61							
PF2	0.59							
PF3	0.74							
PF4	0.76							
PF5	0.71							
PF6	0.67							
PF7				0.56				
PF8				0.87				
PF9				0.78				
PF10	0.49							
RP1		0.77						
RP2		0.85						
RP3		0.79						
RP4		0.78						
BP1							0.62	
BP2							0.88	
GH1					0.30			
GH2			0.35					
GH3								0.64
GH4		0.19						
GH5								0.71
VT1					0.44			
VT2					0.60			
VT3			0.38					
VT4			0.32					
SF1			0.54					
SF2			0.63					
MH1			0.62					
MH2			0.62					
MH3			0.57					
MH4			0.53					
MH5					0.64			
RE1						0.60		
RE2						0.72		
RE3						0.58		

For French version, the eight-factor model obtained by the EFA with varimax rotation accounted for 52% of the common variance. These results are showed in Table 7. All items of the ‘General Health’ (GH), ‘Social Functioning’ (SF) and ‘Bodily Pain’ (BP) subscales each constituted a single factor (resp. 5, 6 and 8). ‘Role Emotional’ (RE) and ‘Role Physical’ subscales were combined into the factor 1. As in the Creole version, the ‘Physical Function’ was divided into 2 factors (resp. 3 and 4) and ‘Mental Health’ (MH) and ‘Vitality’ (VT) items were mixed into two different factors (resp. 2 and 7), but the distribution of the items was different.

**Table 7:** Factor loadings from the exploratory factor analysis on the SF-36 French version.

	Factor1	Factor2	Factor3	Factor4	Factor5	Factor6	Factor7	Factor8
PF1			0.52					
PF2			0.69					
PF3			0.79					
PF4			0.67					
PF5			0.66					
PF6			0.36					
PF7				0.63				
PF8				0.78				
PF9				0.80				
PF10			0.41					
RP1	0.75							
RP2	0.74							
RP3	0.74							
RP4	0.65							
BP1								0.51
BP2								0.47
GH1					0.61			
GH2					0.46			
GH3					0.53			
GH4					0.33			
GH5					0.65			
VT1							0.45	
VT2							0.66	
VT3		0.59						
VT4		0.46						
SF1						0.58		
SF2						0.62		
MH1		0.64						
MH2		0.67						
MH3							0.50	
MH4		0.70						
MH5		0.51						
RE1	0.48							
RE2	0.44							
RE3	0.49							

## Discussion

Our study aimed to translate and culturally adapt the French SF-36 in Creole language following IQOLA method and to evaluate its psychometric properties on patients with type II diabetes in Reunion Island. The SF-36 is the most frequently generic HRQoL used to evaluate patients with type II diabetes (20). It is considered to be a valid, reliable and concise questionnaire. Regarding the psychometric properties of the Creole and French version in this study, we obtained generally, strong internal consistency reliability, consistent convergent validity and good discriminant validity.

Translation and cultural-adaptation methodologies used were similar to those used for the French translation of Leplège A. and al (9). In this study, SF-36 difficulty rating was satisfactory, but clarity of translation was rated as poor compared to other studies that have translated the SF-36 (21–23). However, in these studies, the results of the difficulty and quality ratings varied considerably between countries (21–23). In addition, the Creole version of the SF-36 showed acceptable comprehensibility and face validity.

In contrast to the French SF-36 from Leplège et al. (9), no ceiling and floor effect was found in the present study. The absence of floor or ceiling effects for both the Creole and French versions indicates acceptable measurement standards. Moreover, the internal consistency reliability of the Creole SF-36 was always high, except for the General Health (GH) subscale. In previous studies, a lowest internal consistency reliability was also observed for GH subscale but it remained acceptable (9, 21, 22). These results reflect translator’s feelings, as they indicated more difficulties for translate responses for the GH subscale.

Overall, GH subscale, which measures how patients rate their own overall health status, performed relatively poorly in internal consistency reliability, test–retest reliability, convergent validity and construct validity. In their study translating the SF-36 in Norwegian (22), Loge JH et al., showed a poor convergent validity on 2 items of the subscale GH (GH2 and GH4). They proposed to reassess the translation of these items because GH2 had an ambiguous meaning and could be interpreted as both *“I seem to get sick a little easier than other people”* and *“I seem to get less severely sick than other people”* and GH4 was also somewhat unclear. In our study, Investigators noted that some of GH subscale responses were confusing and that patients hesitated between *“lé vré pou vréman”* (*“definitely true”*) and *“lé vré minm”* (*“mostly true”*) or between *“lé pa vré minm”* (*“mostly false”*) and *“lépa vré ditout”* (*“definitely false”*). The GH subscale in the Creole version should be further investigated.

The test–retest reliability in the Creole version was higher for physical subscales (PF, RP and BP) than for mental subscales (SF, RE, MH). In studies assessing the SF-36 in other diseases, the retest was performed between 2 and 4 weeks after the first questionnaire to obtain acceptable results (23–26). Moreover, in their study, Luscombe et al. demonstrated that type 2 diabetes is frequently associated with negative psychological effects (27). In our study, the retest was realized 4 weeks after the first questionnaire. Therefore, one can think that mental well-being in diabetes patients might vary with time and should be assessed in a shorter time.

Results of discriminant validity were in accordance with the factorial analysis. The structural validity revealed eight factors for both versions, but they were not in fully consistent with hypothetical structure of the SF-36 (6). For this reason, we preferred an EFA than a confirmatory factorial analysis. For instance, we observed, that *“than a mile / several block / one block”* on PF7, PF8, and PF9 questions were perceived by Reunionese population as being redundant. This kind of pattern was observed in Creole and French versions, suggesting that it is not a translation issue. In the original American version, the concept of *“a block”* was used for PF8 and PF9 (6). However, since this concept does not exist in most non–English speaking countries, it was culturally adapted in the translations (28) to express distances in kilometers, which are more adapted in most European countries. For example, “one block” was translated into *“eine Straßenkreuz-ung weit”* (the distance between two street crossings) in the German version (28). In Spain, where urban residents are familiar with the concept of a block but rural residents may not be, items PF8 and PF9 were expressed using both indications of distance, blocks and the metric system (28). Thus, our results suggest that the concepts of *“meters”* and *“kilometers”* are not suitable for the Reunionese population.

The VT subscale was also divided in two factors, for both versions. Conceptually, the vitality items are intended to measure both physical and mental vitality and fatigue (29). For our patients, the Creole SF-36 showed that the states of *“feel worn out / tired”* on VT3 and VT4 questions were related with social functioning and mental health subscales. This result is somewhat similar to that observed by Failde et al. (30), who assessed the validity of the SF-36 in patients with coronary artery disease. They suggested that their patients were associating sadness with lack of vitality and lack of vitality with difficulties in social functioning. In our study, the lack of vitality and social functioning were related to mental health in the Creole version. Same results were observed in the French version for VT and MH subscale. More investigation should be done to understand the vitality concept in Reunionese population.

We also observed in both versions that subscales RP and RE were highly correlated, and in the French version, the RP and RE subscales were consolidated into a unique factor. This result was not reported in the original French translation study (9). However, one study showed this type of result when validating the Mongolian SF-36 (23). In their study, both the RP and RE subscales of the SF-36 were highly correlated with the “Daily Activity” subscale of the COOP/WONCA charts. According to their results, they suggest that Mongolians people recognize that limitations in daily activities are primarily due to physical health problems rather than emotional or mental problems. About 49% of Mongolians people were unemployed, which is comparable to unemployment rates that were observed in our study. However, it should be noted here that Reunionese have different lifestyles and worldviews when than the metropolitan population (31–33). This difference may be a reason why Reunionese consider physical and mental aspects of daily activities as a unified concept.

### Strengths and limitations

To the best of our knowledge, this is the first time that the SF-36 has been validated in a sample of creole-speaking patients. This validation of the scale in Creole is preliminary, and other psychometric properties should be evaluated. One limitation is that our study was conducted on a specific population of type II diabetes population on Reunion Island, which may limit the generalizability of the findings to other populations. Moreover, the retest has not been done for the French version. However, this study shows that the SF-36 is a reliable tool in measuring HRQoL of type II diabetes patients. The tool can be explored further to assess the quality of life of the Reunion population and to compare it with patients suffering from chronic diseases.

## Conclusion

Overall, our findings provided evidence that the SF-36 is suitable for type II diabetes patients in Reunion Island in both Creole and French versions. Indeed, both have overall the same psychometrics properties. Investigators felt the French version was easier to administrate than the Creole version. Further research could be conducted to investigate French–Creole differences in perceived health status and a cultural adaptation of the French version will be considered.

## Data availability statement

The raw data supporting the conclusions of this article will be made available by the authors, without undue reservation.

## Ethics statement

The studies involving human participants were reviewed and approved by Comité de protection des personnes (CPP). The patients/participants provided their written informed consent to participate in this study.

## Author contributions

IS: formal analysis and writing – original draft. CF: supervision, validation, visualization, and writing – review and editing. LB, MS, and BF: visualization and writing – review and editing. XD: resources. SL: project administration. LH: methodology, supervision, and funding acquisition. AT: writing – final draft. All authors contributed to the article and approved the submitted version.

## Funding

This study was supported by the “Appel à Projets Interrégional sur la recherche clinique ou en population dans l’environnement ultramarin, APIDOM” 2013 of the French Ministry of Health (Groupement Interrégional de Recherche Clinique et d’Innovation Sud-Ouest Outre-Mer Hospitalier, GIRCI SOHO). The Centre Hospitalier Universitaire de la Réunion is the key sponsor of this study and by delegation the Department of Clinical Research and Development supervises all work in accordance with the French public health code.

## Conflict of interest

The authors declare that the research was conducted in the absence of any commercial or financial relationships that could be construed as a potential conflict of interest.

## Publisher’s note

All claims expressed in this article are solely those of the authors and do not necessarily represent those of their affiliated organizations, or those of the publisher, the editors and the reviewers. Any product that may be evaluated in this article, or claim that may be made by its manufacturer, is not guaranteed or endorsed by the publisher.
